# Detection of Biofilm Production and Antibiotic Susceptibility Pattern among Clinically Isolated *Staphylococcus aureus*

**DOI:** 10.1155/2024/2342468

**Published:** 2024-05-06

**Authors:** Sushant Pokhrel, Namrata Sharma, Suraj Aryal, Rachita Khadka, Tika Bahadur Thapa, Pawan Pandey, Govardhan Joshi

**Affiliations:** ^1^Department of Laboratory Medicine, Manmohan Memorial Institute of Health Sciences, Kathmandu, Nepal; ^2^Department of Microbiology, Nobel College, Kathmandu, Nepal; ^3^Department of Microbiology, Chitwan Medical College, Bharatpur, Nepal; ^4^Department of Pharmacology, Nobel College, Kathmandu, Nepal; ^5^Department of Microbiology, Global Hospital, Lalitpur, Nepal

## Abstract

**Aim:**

The increasing antibiotic resistance and the ability to form biofilms in medical devices have become the leading cause of severe infections associated with *Staphylococcus aureus* (*S. aureus*). Since the bacteria living in biofilms can exhibit 10- to 1,000-fold increase in antibiotic resistance and implicate chronic infectious diseases, the detection of *S. aureus* ability to form biofilms is of great importance for managing, minimizing, and effectively treating infections caused by it. This study aimed to compare the tube and tissue culture methods to detect biofilm production and antibiotic susceptibility in MRSA and MSSA.

**Materials and Methods:**

The *S. aureus* isolates were identified by the examination of the colony morphology, Gram staining, and various biochemical tests. Antimicrobial susceptibility testing of all isolates was performed by the modified Kirby–Bauer disc diffusion method as recommended by CLSI guidelines. MRSA screening was performed phenotypically using a cefoxitin disc (30 *µ*g). Isolates were tested for inducible resistance using the D-test, and two phenotypic methods detected biofilm formation.

**Results:**

Among 982 nonrepeated clinical specimens, *S. aureus* was isolated from 103 (10.48%). Among 103 clinical isolates of *S. aureus*, 54 (52.42%) isolates were MRSA, and 49 (47.57%) were MSSA. Among 54 MRSA isolates, the inducible MLSB phenotype was observed in 23/54 (42.59%) with a positive D-test. By TCP method, 26 (48.1%) MRSA isolates were strong biofilm producers, whereas, among all MSSA isolates, only 6 (12.2%) were strong biofilm producers.

**Conclusion:**

MRSA showed strong biofilm production in comparison with MSSA. The TCP method is a recommended reliable method to detect the biofilm among *S. aureus* isolates, and the TM method could be useful for the screening of biofilm production in *S. aureus* in the routine clinical laboratory.

## 1. Introduction

*Staphylococcus aureus*, a virulent human pathogen, is the most known cause of community and nosocomial infections. Almost 41% of the general population carries *S. aureus,* colonizing the upper respiratory tract asymptomatically as a human microbiota, which might serve as a source for invasive infections [1], resulting in minor to severe systemic infections such as pulmonary infections, and infective endocarditis, leading to treatment failures and death [2, 3]. With the improper use of multiple drugs and development of resistance to the drugs, their ability to form biofilm, *S. aureus* is capable of causing diverse infections not responsive to the usual therapy [4]. Bacteria living as a biofilm generally show increased resistance to antibiotics and can evade the host immune system acting in the complement cascade, impairing deposition of C3b, key complement component, and C3b opsonization, complicating the effectiveness of antimicrobial therapy and clearance from the body [5].

The *S. aureus* isolates resistant to methicillin were first described in 1961, shortly after the introduction of methicillin in clinical practice; however, MRSA strains were present even before the introduction of methicillin, which was thought to be due to the use of penicillin previously [6]. Furthermore, the irrational use of conventionally available antibiotics and ability of forming biofilms has increased antibiotic resistance among S. aureus and MRSA isolates often show multidrug resistance (MDR), resistance to *β*-lactam antibiotics and tetracyclines, macrolide, chloramphenicol, and fluoroquinolones commonly used in the treatment and management of S. aureus infection [7].

The structured community of bacterial cells enclosed in a self-produced polymeric matrix is defined as a biofilm that is adherent to an inert or living surface [8]. A biofilm in *S. aureus* is generally associated with the expression of polysaccharide intercellular adhesion (PIA) protein, which is a poly-*β*(1–6)-N-acetylglucosamine (PNAG). PIA is responsible for cell aggregation and cell-to-cell adhesion and is encoded by *ica* operon (*ica*ADBC) [9]. The increasing use of catheters, endotracheal tubes, prosthetic devices, etc. has increased the risk of bacterial infections like staphylococcal infections associated with biofilm formation in medical devices, which could be the reason for increased morbidity, mortality, and socioeconomic burden [10]. Hence, the detection and differentiation of *S. aureus* and its ability to form a biofilm in such devices are of great importance for managing, minimizing, and effectively treating such infections [11]. Various phenotypic methods such as the tissue culture plate method (TCP), Congo-red agar method (CRA), tube method (TM), and electron microscopy are available for the detection of the biofilm. However, the TCP is quantitative and considered the gold standard for biofilm detection, whereas CRA and TM are qualitative methods [12].

## 2. Materials and Methods

This laboratory-based cross-sectional study was conducted from July 2020 to December 2020 at the Department of Microbiology, Global Hospital, Lalitpur, Nepal.

### 2.1. Inclusion Criteria

Samples collected from patients of all age groups and genders visiting the hospital during the study period with suspected infections were included in the study.

### 2.2. Exclusion Criteria

Repeated samples, samples collected after antibiotic therapy, and samples showing signs of contamination were excluded from the study.

### 2.3. Sample Processing and Identification of *S. aureus*

A total of 982 nonrepeated clinical specimens collected from July 2020 to December 2020 were included in the study. All the samples were processed in the microbiology laboratory following the standard microbiological procedures. Initially, all the samples were streaked on blood agar and mannitol salt agar plates and incubated aerobically at 37°C for 24 hr. The isolates were first identified as *staphylococcal* strains depending on the colony morphology and Gram staining. The yellow-colored colonies on mannitol salt agar and cream to golden yellow colonies with *β* or weak hemolysis on blood agar were subcultured on nutrient agar (NA) and incubated aerobically at 37°C for 24 hr. After incubation, the isolated colonies from NA plates were used for biochemical tests including catalase, coagulase (slide and tube coagulase test), and DNase tests for the identification of *S. aureus* isolates.

### 2.4. Antimicrobial Susceptibility Testing

Antimicrobial susceptibility testing of all isolates of *S. aureus* was performed by modified Kirby–Bauer disc diffusion method on Mueller–Hinton Agar (MHA) (HiMedia Pvt. Ltd. India), as recommended by CLSI guidelines [13]. Isolates were tested for susceptibility against amikacin (30 *µ*g), gentamicin (10 *µ*g), ofloxacin (5 *µ*g), clindamycin (2 *µ*g), erythromycin (15 *µ*g), ceftazidime (30 *µ*g), linezolid (30 *µ*g), levofloxacin (5 *µ*g), ampicillin (10 *µ*g), cefoxitin (30 *µ*g), cotrimoxazole (25 *µ*g), ampicillin-clavulanate (10 *µ*g) and vancomycin (30 *µ*g) (HiMedia Pvt. Ltd. India). The results were interpreted as per the guidelines of the CLSI zone size interpretive chart.

### 2.5. Phenotypic Detection of MRSA

All isolates of *S. aureus* were screened for MRSA phenotypically using a cefoxitin disc (30 *µ*g) (HiMedia Pvt. Ltd. India). Isolates were lawn cultured on MHA, a cefoxitin disc was placed, and the plates were incubated aerobically at 37°C for 24 hr. *S. aureus* yielding a zone diameter less than 21 mm with cefoxitin disc were phenotypically confirmed as MRSA [14].

### 2.6. Phenotypic Detection of Inducible Clindamycin Resistance (iMLSB Phenotypes)

**Figure 1:**
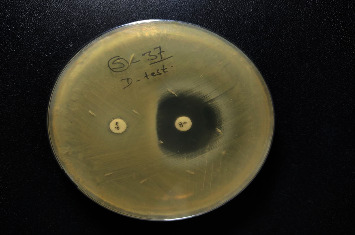
Phenotypic detection of inducible clindamycin resistance by the D-test.

The *S. aureus* isolates showing resistance to erythromycin were tested for inducible resistance to clindamycin. In addition, isolates were tested for inducible resistance using the D-test as per the CLSI guidelines. In brief, 0.5 McFarland standard bacterial suspension was lawn cultured on MHA plates on which the erythromycin disc (15 *µ*g) and clindamycin disc (2 *µ*g) were placed 15 mm apart from edge to edge. The plates were incubated aerobically at 37°C for 24 hr; if the isolate was resistant to erythromycin (zone diameter ≤13 mm) and susceptible to clindamycin (zone diameter ≥21 mm) with a D-shaped zone around clindamycin, it was considered positive for inducible clindamycin resistance [3] (Figure 1).

### 2.7. Biofilm Formation Assay

Biofilm formation was detected by in vitro methods: Tube method (TM) and tissue culture method (TCP). Strong biofilm-producing *S. epidermidis* strain ATCC 35984 was used as a positive control in both the tests performed.

### 2.8. Tube Method

**Figure 2:**
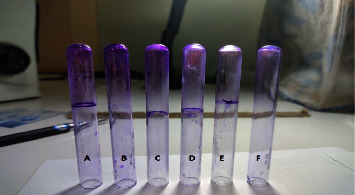
Biofilm formation assay by tube method (TM). (A) Positive control (PC); (B) strong biofilm producer; (C) moderate biofilm producer; (D) weak biofilm producer; (E) nonbiofilm producer; (F) negative control.

Biofilm detection using the tube method was performed as per Christensen et al. [15]. Briefly, a loop full of inoculum from overnight culture plates was inoculated in 5 ml trypticase soy broth (TSB) with 1% glucose and incubated overnight at 37°C. The tubes were emptied following incubation, washed with phosphate buffer saline (PBS) pH 7.2, air-dried, and stained with 0.1% crystal violet followed by washing with deionized water to remove excess stain. The tubes were air-dried and observed visually for biofilm production. Biofilm production was indicated by the formation of a visible film lining the wall and bottom of the tube while ring formation at the liquid air interface was not considered biofilm production. The results were categorized in reference to the control strain as weak/nonproducers, moderate, and strong biofilm producers, depending upon the intensity of the film observed visually (Figure 2).

### 2.9. Tissue Culture Plate (TCP) Method

Biofilm detection using the TCP method was performed as per Christensen et al. [16]. Isolates of *S. aureus* from the new culture were inoculated in 2 ml TSB with 1% glucose and incubated overnight at 37°C. 1 : 100 dilution was prepared with fresh TSB medium, and 200 *µ*l of dilution was inoculated into individual wells of sterile, flat-bottom polystyrene tissue culture plates. At the same time, *S. epidermidis* strain ATCC 35984 was also processed simultaneously, which served as a positive control, and un-inoculated wells, which served as a negative control. The plates were incubated overnight at 37°C. Following incubation, the contents of each well were removed by pipetting and tapping the plates gently followed by washing with 200 *µ*l PBS (pH 7.3) to remove free-floating bacteria. Biofilms adherent to wells were fixed with 2% sodium acetate and stained with 0.1% crystal violet. The wells were washed gently with deionized water and air-dried followed by the determination of the optical density of the stained adherent biofilm using an ELISA reader (HumaReader HS, Human Diagnostics, Germany) at 570 nm. The experiments were performed in triplicate for each well. Biofilm production was categorized as weak/nonbiofilm producers, moderate, and strong biofilm producers based on the optical density measured and compared with that of control strain as mentioned by Abdel Halim et al. (Table 1) [17].

### 2.10. Data Analysis

Each sample was encoded with an identification number, and findings were manually recorded and entered into Microsoft Excel 2016 and analyzed using SPSS version 20.0 (IBM Corp., Armonk, NY, USA). Findings were expressed in terms of number and percentage.

## 3. Results

A total of 982 nonrepeated clinical specimens were processed in the microbiology laboratory, of which *S. aureus* was isolated from 103 (10.48%) clinical specimens. Among 103 clinical isolates of *S. aureus*, 54 (52.42%) isolates were MRSA and 49 (47.57%) were MSSA.

Among different clinical specimens processed, *S. aureus* mainly were isolated from wound/pus swab 63 (61.16%), followed by urine 21 (20.38%), blood 7 (6.79%), catheter tips 7 (6.79%), and others 5 (4.85%), respectively. Among 63 isolates of *S. aureus* isolated from Wound/pus swabs, 35 (55.55%) were MRSA, and 28 (44.44%) were MSSA. Similarly, 9 (42.85%) isolates were MRSA, and 12 (57.14%) isolates were MSSA among 21 clinical isolates of *S. aureus* isolated from urine (Table 2).

### 3.1. Antibiotic Susceptibility Pattern of *S. aureus*

Among 103 clinical isolates of *S. aureus*, 54 were methicillin-resistant, whereas 49 were methicillin-sensitive. Most of the strains were resistant to ceftazidime (76.7%) and ampicillin-clavulanate (71%), followed by ofloxacin (63%) and cotrimoxazole (59.2%). In addition, 24.3% of isolates were resistant to vancomycin. However, almost all (99%) isolates were sensitive to linezolid and similarly higher sensitivity was seen for amikacin (98%), followed by clindamycin (80.6%) and vancomycin (75.7%) (Table 3).

### 3.2. Inducible Clindamycin Resistance Pattern of *S. aureus*

Among 103 *S. aureus* isolates, 10 (9.7%) were resistant to erythromycin and clindamycin, whereas 20 (19.41%) were sensitive to erythromycin and clindamycin. Furthermore, 42 (40.77%) isolates that were sensitive to clindamycin and resistant to erythromycin showed positive D-test, indicating the inducible MLSB phenotype, whereas 31 (30%) isolates showed true clindamycin sensitivity, giving negative D-test, indicating macrolide sensitive phenotype. Among MRSA, the inducible MLSB phenotype was observed in 23/54 (42.59%), while in MSSA, it was observed in 19/49 (38.77%). It showed a higher iMLSB resistance in MRSA than in MSSA strains (Table 4).

## 4. Biofilm Production among MRSA and MSSA

In vitro biofilm production was measured using two different phenotypic assays: TM and TCP. Among 54 MRSA strains, 19 (35.2%) were strong biofilm producers, whereas 24 (44.44%) were moderate biofilm producers, and 11 (20.4%) were nonbiofilm producers in the TM method. Similarly, among MSSA, 18 (36.7%) were strong, 15 (30.6%) were moderate, and 16 (32.7%) were nonbiofilm producers in the TM method.

**Figure 3:**
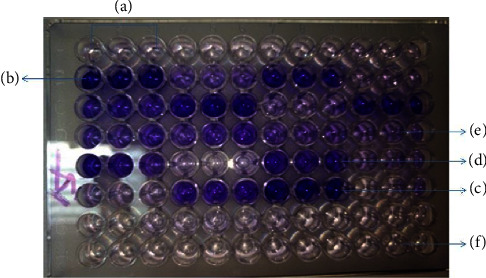
Biofilm formation assay by the tissue culture plate (TCP) method. (a) Negative control; (b) positive control; (c) strong biofilm producer; (d) moderate biofilm producer; (e) weak biofilm producer; (f) nonbiofilm producer.

By the TCP method, 26 (48.1%) MRSA isolates were strong biofilm producers, and 18 (33.3%) were moderate biofilm producers, whereas among all MSSA isolates, only 6 (12.2%) were strong and 10 (20.4%) were moderate biofilm producers. Of a total of 54 MRSA isolates, 10 (18.5%) and, among 49 MSSA isolates, 33 (67.3%) were weak or nonbiofilm producers (Table 5) (Figure 3).

## 5. Discussion

*Staphylococcus aureus*, the common cause of community and healthcare-associated infections, is capable of causing multiple acute and chronic infections such as pulmonary infections and infective endocarditis. Colonization of *S. aureus* as biofilms in nasopharynx serves as a reservoir for local and invasive diseases. A recent study suggested *S. aureus* and *Streptococcus pneumoniae* can cocolonize the nasopharynx forming the dual species biofilms and *S. pneumoniae* modulates *S. aureus* biofilm dispersion and transition from colonization to invasive disease. Additionally, physiological changes in response to certain infections like influenza A virus (IAV) promote the dispersal of bacteria from the biofilms and disseminate from colonized nasal tissue to the lungs [18]. Similarly, the ability of *S. aureus* to form biofilms in indwelling medical devices such as catheters and prostheses and increasing antibiotic resistance has led to the severe infections associated with *S. aureus* not responsive to usual therapy. Therefore, timely and early detection of biofilm production among *S. aureus* highlights the vital steps to prevent, manage, and adequately treat infections caused by it [2]. Furthermore, the effective and reliable diagnostic method is essential in healthcare settings for the timely management of biofilm-associated staphylococcal infections [19]. Therefore in this study, we examined biofilm formation by two commonly and routinely used phenotypic methods of in vitro biofilm detection.

In this study, phenotypic methods tested 103 clinical isolates of *S. aureus* isolated from different clinical samples for antibiotic sensitivity and biofilm production. Among 103 clinical isolates of *S. aureus*, 54 (52.42%) isolates were MRSA, and 49 (47.57%) were MSSA. Our study showed a slightly higher incidence of MRSA isolates compared to the previously published reports: 43.1%, 45.9%, and 47.4% of MRSA isolates from Nepal [20–22]. The incidence of MRSA seems to be increasing in Nepal as the recently published reports by Sapkota et al. (70.6%) [23] and Dhungel et al. (87.2%) [24] have reported higher cases of MRSA than in our study.

In our study, we found that 94.4% of MRSA isolates were resistant to ceftazidime, which is in accordance with the study performed by Hussaini et al., where they found that 100% of MRSA isolates were resistant to ceftazidime [25]. Similarly, the higher rate of resistance among MRSA isolates was with ampicillin-clavulanate (89%), followed by ofloxacin (77.8%). Resistance to cotrimoxazole among MRSA isolates was found to be 74%, which is higher as compared to 63.2% reported by Belbase et al. [20] and 55.9% reported by Manandhar et al. [2]. Furthermore, in contrast to 32.7% erythromycin resistance noted by Ansari et al. [21] and 55.3% noted by Belbase et al. [20], we reported a higher rate (64.8%) of resistance to erythromycin among MRSA isolates. In contrast to the previously published studies, we found that 29.7% of MRSA were resistant to vancomycin [20]. In accordance with our findings, a study performed by Goswami et al. in India reported that 45.83% of *S. aureus* isolates were resistant to vancomycin. The study also suggested that the resistance of *S. aureus* to vancomycin should be interpreted cautiously as it could be a false-positive result due to the contamination with bacteria like *Acinetobacter* and also mentioned that resistance to vancomycin among MRSA isolates is an alarming situation [26].

In our study, higher rates of multidrug resistance were found among MRSA isolates due to the various risk factors contributing to resistance in developing countries like Nepal. The lack of awareness among the public about the proper use of antibiotics, availability of antibiotics without prescription and prescription by unauthorized personnel, self-medication by the patient without consulting the doctor, improper dosage of antibiotics, and incomplete courses of antibiotics use, and the lack of proper facilities in the laboratories to detect antibiotic resistance could explain the increasing rate of antibiotic resistance among clinical isolates in Nepal [21]. Since the increasing rate of antibiotic resistance and biofilm-forming ability among *S. aureus* is worrisome, possible alternative treatments are of great concern. Recently, Sempere et al. [27] have identified that antioxidants such as N-acetyl-L-cysteine (NAC) and cysteamine (Cys) can be the promising drug candidates against individual or mixed *S. aureus* biofilms. Additionally, the higher sensitivity of linezolid, amikacin, and clindamycin among MRSA and MSSA isolates in this study indicates these antibiotics could be used as an option for the preliminary treatment of *Staphylococcal* infections.

Furthermore, we detected inducible clindamycin resistance in 40.77% of isolates, which is significantly higher in comparison with previously published reports [28–30], and in accordance with the previously published reports from Nepal [29, 30], it was higher among MRSA isolates (42.5%) as compared to MSSA isolates (38.7%). With the emergence of resistance among *S. aureus* to multiple antibiotics, the use of reserve drugs (like the MLSB family) is being opted for the management of *S. aureus* infection. However, among the MLSB family, clindamycin is the better choice due to its lower side effects and better tissue penetration capability. And during clindamycin therapy, iMLSB strains can gradually develop constitutive or inducible clindamycin resistance, leading to treatment failure in some patients. Hence, to minimize treatment failures, detecting such resistant phenotypes is essential in routine practice [31]. Therefore, these findings indicate that the D-test is vital in routine laboratories for the preliminary identification of inducible clindamycin resistance, which could effectively manage and minimize treatment failures that are likely to occur.

In our study, biofilm formation was detected using two different phenotypic assays, i.e., TM and TCP. As mentioned by Hassan et al., the TCP method is the gold standard method for the detection of biofilm [32, 33]. Since there is the use of multiple controls and the experiments being performed in triplets, TCP provides better assessment of *S. aureus* biofilms. Similarly, Manandhar et al. mentioned that the best correlation was shown by the TCP method with the presence of icaAD genes [2]; hence, we considered it as a standard method for the interpretation of our results. With the TCP method, strong biofilm production was significantly higher among MRSA isolates (48.1%) in comparison with MSSA isolates (12.2%), which is in accordance with the findings of Manandhar et al. [2]. Similarly, Piechota et al. in Poland also found a higher rate of biofilm formation among MRSA isolates than MSSA isolates [7]. A higher rate of biofilm formation among MRSA isolates indicates biofilm detection in routine laboratories for managing antibiotic resistance and minimization of treatment failure.

Due to resource and time limitations, we could not perform the molecular testing of methicillin resistance and biofilm formation among *S. aureus* isolates, and the minimum inhibitory concentration test for vancomycin and the study was limited to a single center only.

## 6. Conclusion

The present result demonstrates that MRSA and MSSA showed biofilm production; however, MRSA showed strong biofilm production compared to MSSA. Biofilm production results in resistance to antibiotics in *S. aureus*. Early detection of biofilm production in clinically isolated *S. aureus* mitigates the use of resistant antibiotics in patients. TCP is a recommended reliable method to detect biofilm production among *S. aureus*, additionally TM could be useful method in routine screening of biofilm production among *S. aureus* isolates in a resource-limited settings. Therefore, we recommend screening the biofilm production in *S. aureus* in the routine clinical laboratory.

## Figures and Tables

**Table 1:** Distribution of OD value in biofilm formation.

OD value	Biofilm formation
<0.120	Nonbiofilm producer
0.120–0.240	Moderate biofilm producer
>0.240	Strong biofilm producer

**Table 2:** Distribution of bacterial isolates.

Clinical specimen	MRSA	MSSA	Total
	*N* (%)	*N* (%)	*N* (%)
Wound/Pus swab	35 (55.55)	28 (44.44)	63 (61.16)
Urine	9 (42.85)	12 (57.14)	21 (20.38)
Blood	0 (0)	7 (100)	7 (6.79)
Catheter tips	7 (100)	0 (0)	7 (6.79)
Others	3 (60)	2 (40)	5 (4.85)
Total	54 (52.42)	49 (47.57)	103 (100)

**Table 3:** Antibiotic susceptibility pattern of bacterial isolates.

Antibiotics	MSSA (*N* = 49)		MRSA (*N* = 54)		Total (*N* = 103)	
	Sensitive	Resistant	Sensitive	Resistant	Sensitive	Resistant
	*N* (%)	*N* (%)	*N* (%)	*N* (%)	*N* (%)	*N* (%)
Ampicillin (AMP)	15 (30.7)	34 (69.3)	—	—	15 (30.7)	34 (69.3)
Cefoxitin (CX)	49 (100)	0 (0)	0 (0)	54 (100)	49 (47.5)	54 (52.4)
Ceftazidime (CAZ)	21 (42.8)	28 (57.2)	3 (5.6)	51 (94.4)	24 (23.3)	79 (76.7)
Ampicillin-clavulanate (AMPC)	24 (49)	25 (51)	6 (11)	48 (89)	30 (18)	73 (71)
Gentamicin (GEN)	34 (69.3)	15 (30.7)	24 (44.4)	30 (55.6)	58 (56.3)	45 (43.7)
Amikacin (AK)	49 (100)	0 (0)	52 (96.3)	2 (3.7)	101 (98)	2 (2)
Cotrimoxazole (COT)	28 (57.2)	21 (42.8)	14 (19)	40 (74)	42 (40.8)	61 (59.2)
Ofloxacin (OF)	26 (53)	23 (47)	12 (22.2)	42 (77.8)	38 (37)	65 (63)
Levofloxacin (LE)	30 (61.3)	19 (38.7)	16 (29.7)	38 (70.3)	46 (44.7)	57 (55.3)
Clindamycin (CD)	39 (79.6)	10 (20.4)	44 (81.5)	10 (18.5)	83 (80.6)	20 (19.4)
Erythromycin (E)	26 (53)	23 (47)	19 (35.2)	35 (64.8)	45 (43.7)	58 (56.3)
Linezolid (LZ)	49 (100)	0 (0)	53 (98.2)	1 (1.8)	102 (99)	1 (1)
Vancomycin (VA)	40 (81.6)	9 (18.4)	38 (70.3)	16 (29.7)	78 (75.7)	25 (24.3)

**Table 4:** Inducible clindamycin resistance in MRSA and MSSA.

Phenotypes	MRSA	MSSA	Total
	*N* (%)	*N* (%)	*N* (%)
E-S, CD-S	11 (20.3)	9 (18.3)	20 (19.4)
E-R, CD-R (constitutive MLSB)	7 (12.9)	3 (6.1)	10 (9.7)
E-R, CD-S (D+) (Inducible MLSB)	23 (42.5)	19 (38.7)	42 (40.7)
E-R, CD-S (D−) (MS)	13 (24.1)	18 (36.7)	31 (30.1)
Total	54 (100)	49 (100)	103 (100)

E: erythromycin; CD: clindamycin; S: sensitive; R: resistance; (D+): D-test positive; (D−): D-test negative; MS: macrolide sensitive.

**Table 5:** Biofilm production among MRSA and MSSA.

Biofilm production	TM method		TCP method	
	MRSA, *n* (%)	MSSA, *n* (%)	MRSA, *n* (%)	MSSA, *n* (%)
Strong (+++)	19 (35.2)	18 (36.7)	26 (48.1)	6 (12.2)
Moderate (++)	24 (44.4)	15 (30.6)	18 (33.3)	10 (20.4)
Weak/None (±)	11 (20.4)	16 (32.7)	10 (18.5)	33 (67.3)

## Data Availability

All the data generated during this study are presented in this paper. The primary raw data will be made available from the corresponding author upon reasonable request.
